# 

**Published:** 1896-08

**Authors:** 


### CONTENTS:

### SELECTIONS.

Article I.—Tobacco from an Esthetic and Hygienic Point of View. By H. M. Ochiltree, M. D. - - - 145
" II.—Chlorate of Potash as an Antiseptic and Germicide for the Mouth. - - - - - - 156
" III.—Practical Laboratory Points. By J. G. Templeton, D. D. S., - - - - - - 159
" IV.—Solid Cusps. By Dr. Cephas Whitney. - - 161
" V.—Soldering. By Dr. H. J. Goslee. - - - 164
" VI.—Abstract From a Discussion Following the Reading of a Paper Upon "Soft Foil and Conservative Methods." By Dr. W. T. Arrington. - - 171
" VII.—Implantation. - - - - - - 178

### COMMUNICATED.

New Jersey State Dental Society. - - - - - - 182

### MONTHLY SUMMARY.

Skiagraphy in Dentistry. - - - - - - - 182

Alloy and Cement for Filling. - - - - - - 183

Dr. Genese on an Efficient Obtundent. - - - - 184

New Treatment for Pyorrhea. - - - - - - - 184

Amalgam Filling. - - - - - - - - 185

Local Anesthetic. - - - - - - - - 186

Percentage Solutions. - - - - - - - 186

Amalgamating Gold Sections into Teeth. - - - - 186

Root Canal Cleansers. - - - - - - - 187

Treatment of Root Canals for Immediate Filling. - - - 188

To Bore Glass. - - - - - - - - 188

The House We Live In. - - - - - - - 189

Nerve Tension. - - - - - - - - 189

The Truth About Bacteriology. - - - - - - 190

Calomel Treatment in Typhoid Fever. - - - - 191

A Cheap Hydrogen-gas Flash Lamp. - - - - - 192

Treating Dead Teeth. - - - - - - - 192

Cataphoresis. - - - - - - - - 192

In Anesthesia. - - - - - - - - 192

